# Biochemical and Structural Analyses Shed Light on the Mechanisms of RadD DNA Binding and Its ATPase from *Escherichia coli*

**DOI:** 10.3390/ijms24010741

**Published:** 2023-01-01

**Authors:** Li-Fei Tian, Xiaolin Kuang, Ke Ding, Hongwei Gao, Qun Tang, Xiao-Xue Yan, Wenqing Xu

**Affiliations:** 1 National Laboratory of Biomacromolecules, CAS Center for Excellence in Biomacromolecules, Institute of Biophysics, Chinese Academy of Sciences, Beijing 100101, China; 2 College of Life Sciences, University of Chinese Academy of Sciences, Beijing 100049, China; 3 School of Life Science and Technology, ShanghaiTech University, Shanghai 201210, China

**Keywords:** DNA repair, ATPase, helicase superfamily 2, SSB protein, DNA binding, crystal structure

## Abstract

DNA double-strand breaks (DSBs) are the most perilous and harmful type of DNA damage and can cause tumorigenesis or cell death if left repaired with an error or unrepaired. RadD, a member of the SF2 family, is a recently discovered DNA repair protein involved in the repair of DSBs after radiation or chemical damage. However, the function of RadD in DNA repair remains unclear. Here, we determined the crystal structures of RadD/ATPγS and RadD/ATP complexes and revealed the novel mechanism of RadD binding to DNA and ATP hydrolysis with biochemical data. In the RadD catalytic center, the Gly34 and Gly36 on the P-loop are key residues for ATP binding besides the conserved amino acids Lys37 and Arg343 in the SF2 family. If any of them mutate, then RadD loses ATPase activity. Asp117 polarizes the attacking water molecule, which then starts a nucleophilic reaction toward γ-phosphate, forming the transition state. Lys68 acts as a pocket switch to regulate substrate entry and product release. We revealed that the C-terminal peptide of single-stranded DNA-binding protein (SSB) binds the RadD C-terminal domain (CTD) and promotes the RadD ATPase activity. Our mutagenesis studies confirmed that the residues Arg428 on the zinc finger domain (ZFD) and Lys488 on the CTD of RadD are the key sites for binding branched DNA. Using the Coot software combined with molecular docking, we propose a RadD-binding DNA model for the DNA damage repair process.

## 1. Introduction

The stability of cellular DNA is crucial for the transmission of life. DNA replication occurs all the time, and the DNA may be damaged by some endogenous or exogenous sources. Organisms have evolved a series of DNA damage response mechanisms to repair various types of DNA damage, thereby ensuring DNA stability and life reproduction. The radD (yejH) gene from *Escherichia coli* (*E. coli*) has been recently found to have an unspecified role in repairing radiation damage, particularly DNA double-strand breaks (DSBs) [1,2].

RadD is classified into the helicase superfamily (SF) 2, which contains all seven conserved motifs of SF2 and a small homology sequence outside of these motifs [1]. SF2 is the largest and most diverse of the SFs and is divided into 10 families, namely, DEAD-box RNA helicases, DEAH/RHA, RecQ-like, Rad3/XPD, Swi/Snf, RIG-I-like, Type I Restriction Enzyme, Ski2-like, RecG-like and NS3/NPH-II [3,4]. SF2 helicases are involved in DNA repair [5,6], chromatin rearrangement [7], transcription [8], and RNA metabolism [9]. In Vibrio cholera, RadD clears RNA polymerase from UV-induced DNA lesions [10,11]. A recent study showed that RadD is an accessory protein that accelerates RecA-mediated DNA strand exchange when RecA is present only [12].

RadD has DNA-independent ATPase activity. ATPases contain different numbers of RecA-like domains (RDs). Two RDs in a single polypeptide chain are found in many SFs 1 and 2 [13]. RD1 contains motifs Q, I, Ia, II, and III, and RD2 contains motifs IV, V, and V. ATP binding sites are speculated in accordance with the structural model of RadD/AMPPNP [14] and RadD/ADP [15]. The conserved Lys37 and Arg343 interact with β- and γ-phosphoric acids of ATP, respectively [14,15], and Lys68 on the motif Ia shows binding to the ATP molecule [14], which is not reported in other SF2 helicases.

The bacterial single-stranded DNA-binding protein (SSB) interacts with many members of SF2, which are remarkable for all aspects of cell metabolism [16] and is an important organizer in DNA repair complexes [17,18,19]. Similar to most SSB-interacting proteins, RadD can interact with SSB C-terminal residues (SSBct) in vivo and in vitro [11] and can increase the ATPase activity of RadD. RadD can directly bind single-stranded DNA (ssDNA) and branched DNA. Aided by SSB, RadD acts as a clearing agent by binding to stalled replication forks or DNA repair intermediates [11,20].

Despite extensive studies, the ATP hydrolysis mechanism of RadD and the binding sites of RadD to SSB or DNA remain unclear, and the function of RadD in DNA repair remains unknown. Here, we reported the crystal structures of *E. coli* RadD/ATPγS and RadD/ATP at 3.0 Å and 2.7 Å, respectively. An ATPγS or ADP molecule was buried between RD1 and RD2. Based on structural comparisons and biochemical experiments, we have uncovered ATP binding sites and the mechanism of hydrolysis. SSBct bound the RadD C-terminal domain (CTD) and promoted the activity of RadD ATPase. Mutant experiments confirmed that the residues on the zinc finger domain (ZFD) and CTD of RadD are key sites for binding to the branched DNA. We have presented the RadD binding DNA model for DNA damage repair.

## 2. Results

### 2.1. Crystal Structure of RadD-Binding ATPγS or ADP

**Figure 1:**
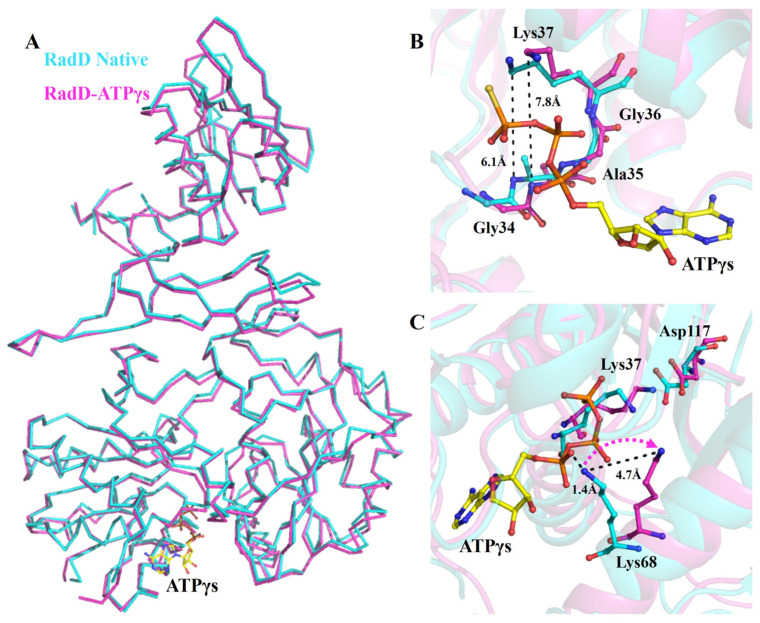
Conformation changes in RadD due to ATP binding. (**A**) Superposition of the structures of apo-RadD and RadD/ATPγS, shown as ribbon. (**B**) Walker A motif expands slightly when RadD binds an ATP, shown as sticks. (**C**) Compared with the structure of apo-RadD, Lys68 shifts away due to the RadD binding the ATP molecule, shown as sticks. Apo-RadD is shown in cyan and RadD/ATPγS in magenta.

In the crystal structure of the RadD/ATPγS complex, an ATP molecule was clearly located in the active site between RD1 and RD2 of RadD. The difference between the overall structures of apo-RadD (our group reported previously) [14] and RadD/ATPγS complex was minimal, and the r.m.s.d. was 0.589 for all C-alpha atoms (Figure 1A). However, sharp differences are observed in the activity center. The distance between Gly34 and Lys37 expanded from 6.1 Å to 7.8 Å after ATPγS binding, and this conformational change made Walker A motif suitable for ATP entry and promoted the formation of hydrogen bonds between β-phosphate of ATP and residues in the P-loop (Figure 1B). This phenomenon was also observed in the complex structure of RecA/ATP [21,22]. In the apo-RadD structure, Lys68 occupied the position of ATP at a distance of 1.4 Å from α-phosphate of ATP but shifted outward by 4.7 Å in the structure of RadD/ATPγS, which provided space for ATP to enter the catalytic center (Figure 1C).

**Figure 2:**
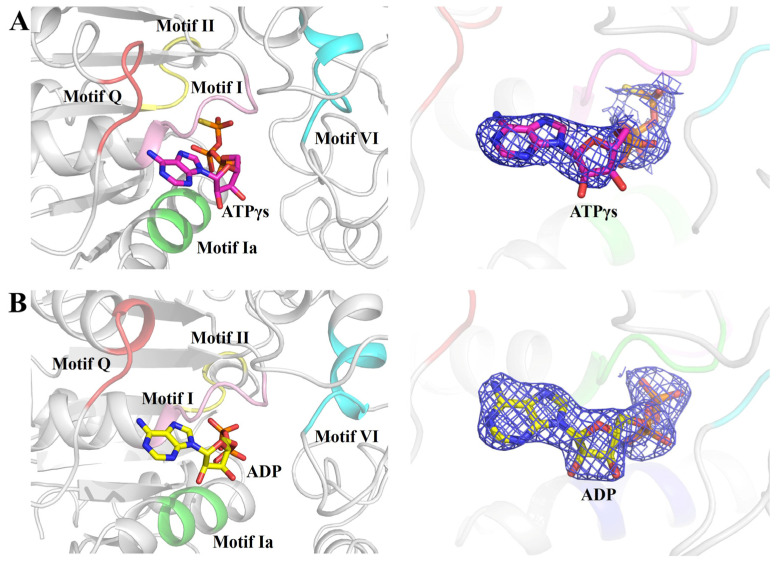
ATPγS or ADP molecule binding in the helicase center (RD1 and RD2) of RadD. (**A**) Enlarged view of five conserved motifs interacting with ATPγS. The motif Q is shown in tv-red, motif I in pink, motif Ia in green, motif II in yellow, and motif VI in cyan. ATPγS is shown as sticks, and the 2Fo-Fc electron density contoured at 1.0 *σ*. (**B**) Enlarged view of five conserved motifs interacting with ADP. ADP is shown as sticks, and the 2Fo-Fc electron density is contoured at 1.0 *σ*.

However, in the structure of the RadD/ATP complex, an ADP molecule was at the same position as ATPγS (Figure 2A,B). RadD/ATP crystals were obtained by incubating and co-crystallizing 5 mg/mL RadD and 5 mM ATP with 5 mM Mg^2+^. The electron density of the structure showed only the density of the ADP molecule and no density of γ-phosphate, indicating that ATP was hydrolyzed into ADP during crystallization.

### 2.2. ATP-Binding Site of RadD

**Figure 3:**
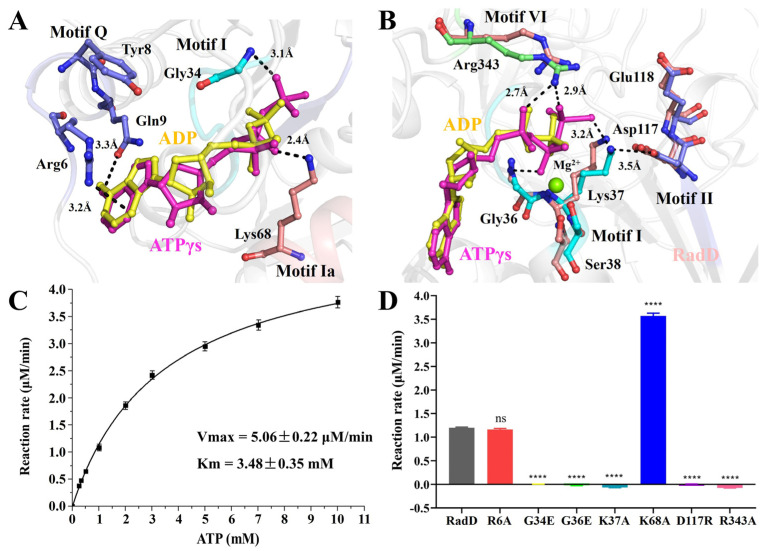
The ATPase activity of RadD. (**A**,**B**) Residues involved in interface interactions between the RadD and ATP molecule. The residues are shown as sticks and the Mg^2+^ ion as a green sphere. (**C**) The ATPase activity shows *Vmax* = 5.06 μM/min and *Km* = 3.48 mM. (**D**) The ATPase activity assays of RadD and mutants at a concentration of 0.375 μM and ATP at a concentration of 1 mM. Error bars indicate the S.D. of at least three independent experiments, **** means *p*-value < 0.0001.

In our RadD/ATPγS structure, five conserved regions interacted with an ATP molecule directly or indirectly (Figure 2A,B). The first region was the SF2 characteristic motif Q (5–9 aa), which interacted with the adenine of ATP. Adenine inserts into the “groove” were formed by Arg6, Tyr8, and Gln9 on motif Q to make the ATP stable. Arg6 NH2 appeared to be H-bonded to adenine N7, and the Gln9 side chain carbonyl formed an H-bond with ATP adenine N6 protons. Tyr8 did not interact directly with the ATP molecule but participated in the formation of “groove” structures (Figure 3A). The second region was the SF2 motif I (33–38 aa), which was also known as P-loop on the Walker A motif. ATP was in the semicircular center, and many pairs of hydrogen bonds were formed amongst the Gly34, Gly36, and Lys37 of the P-loop, which helped the Walker A motif stabilize the position of the ATP molecule (Figure 3B). In addition, Ser38 interacted with an Mg^2+^.

The third region was the SF2 motif Ia (64–68 aa). Lys68 directly formed an H bond with the hydroxyl oxygen atom on the β-phosphate of ATP (Figure 3A). The fourth region was the SF2 motif II, which was also known as the Walker B motif (117–120 aa). Asp117 and Glu118 are conservative negatively charged acidic amino acids within SF characteristic motifs, where Asp117 interacts directly with Mg^2+^ and Glu118 polarizes the attacking water molecule in electron transfer and hydrolysis reactions [23]. However, in our structure of RadD/ATP, Ser38 solidly interacted with the Mg^2+^ ion, Asp117 was away from the Mg^2+^ ion, and Lys37 was in the middle of Asp117 and γ-phosphate of ATP at a distance of 3.2 Å and 3.5 Å, respectively. The side chain of Lys37 and Asp117 moved away from the γ-phosphate of ATP in the structure of RadD/ATP compared with the apo-RadD (Figure 3B). The fifth region was Arg343 in SF2 motif VI (336–343 aa), termed the “arginine finger” in many ATPases. Arg343 approached ATP from the other side, and the N atoms in the side chain of Arg343 formed hydrogen bonds with O atoms on the α- and γ-phosphates of ATP at 2.7 Å and 2.9 Å, respectively (Figure 3B).

### 2.3. ATPase Activity

Based on the structure of the active center, we constructed a series of RadD mutants and examined their ATPase activity. ATPase assay showed RadD WT *Vmax* = 5.06 μM/min and *Km* = 3.48 mM (Figure 3C), but mutant G34E, G36E, K37A, D117R, and R343A lost their ATPase activity (Figure 3D). The mutated G34E and G36E side chains occupy the center of the Walker A motif, preventing ATP from binding to the Walker A motif. Mutants K37A and R343A lost their side chains, resulting in an inability to interact with ATP phosphates. RadD_D117R_ lost the hydrolysis ability of ATPase because Asp117 was replaced by the positively charged acidic amino acid Arg and lost its ability to polarize water molecules. Surprisingly, the ATPase activity of mutant K68A was evidently higher than that of the wild-type RadD (Figure 3D). From the structural analysis, this result can be attributed to the mutation of Lys68 to Gly disrupting the interaction between Lys68 and ATP. ATPase assays showed that mutants R6A and N9A were not significantly different from the wild-type.

**Figure 4:**
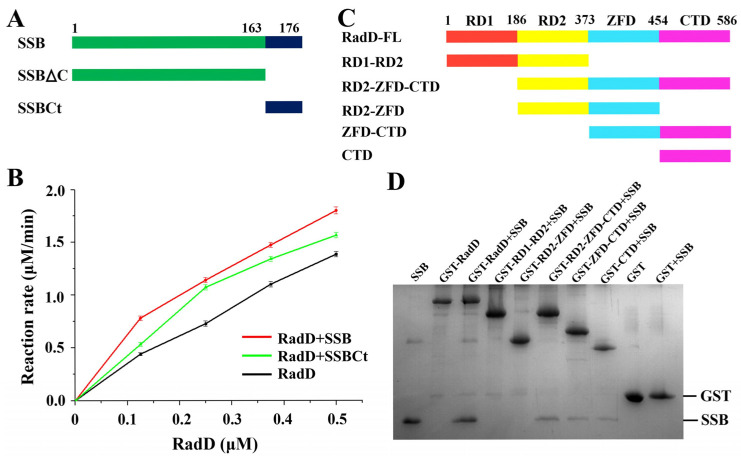
Interaction between RadD and SSB. (**A**) Schematic of SSB domains. (**B**) SSB or SSBct increases the ATPase activity of RadD. (**C**) Schematic of RadD domains. (**D**) Assays of RadD and SSB interactions by GST pull-down. SDS-PAGE is stained using Coomassie Blue.

RadD interacts with SSB in vivo and in vitro, and SSBct is indispensable in the interaction and ATPase activity of RadD [11]. We proved the interaction between RadD and SSB in vitro by molecular exclusion chromatography (Figure S1). A certain concentration of SSB or SSBct was added into RadD and incubated for 30 min, and the ATPase activity of RadD was improved by using ATPase assays (Figure 4A,B). However, the region where RadD interacts with SSB is unknown. We built several truncations of RadD to explore the binding sites of RadD and SSB (Figure 4C). The GST pull-down assay showed that neither RD1-RD2 nor RD2-ZFD were able to pull down the SSB, indicating that RD1, RD2, and ZFD are not interaction regions that bind the SSB. However, D2-ZFD-CTD, ZFD-CTD, and CTD can pull down SSB. Therefore, we defined the CTD as the SSB interaction region (Figure 4D). While the first three domains cannot interact with SSB, they facilitate the interaction between CTD and SSB by stabilizing the structure of RadD.

**Figure 5:**
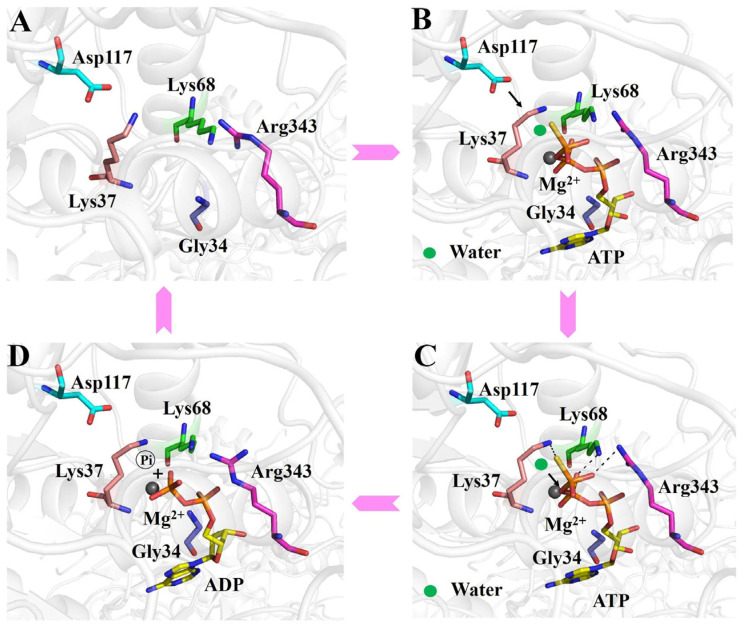
Working mechanism of the ATP binding and hydrolysis of RadD. (**A**) Active center of apo-RadD. (**B**) ATP enters into the active center, and polarization of the attacking water molecule by Asp117. (**C**) Nucleophilic attack, formation, and stabilization of the transition state. (**D**) The chemical bond between γ- and β-phosphoric acids is broken, and the intermediates further become the final products of ADP and phosphoric acid.

Our data support that the ATP hydrolysis process of RadD is divided into the following steps. The phosphate tail of ATP entered the active pocket, and Gly34, Gly36, and Lys37 on the P-loop expanded slightly to accommodate the size of the phosphate group and cooperated with Arg343 on helicase feature motif VI to bind ATP’s β- and γ-phosphoric acids, where hydrogen bonds are formed. Motif Q formed a “groove” structure, which bound the adenine of ATP (Figure 5B). The Walker B motif was close to the ATP molecule, and Ser38 interacted with the Mg^2+^ ion. Asp117 polarized the attack water molecules near the γ-phosphoric acid and initiated a nucleophilic reaction to form a transition state (Figure 5B,C), and then the Walker B motif returned to its original position. Lys37 and Asp117 played an important role in stabilizing the transition state. The chemical bond between γ- and β-phosphoric acids was broken, and the intermediates further became the final products of ADP and phosphoric acid (Figure 5D). ADP exited from the active pocket. The Walker A motif and the relative positions of key amino acids Lys37, Lys68, and Arg343 were slightly tightened, and the active pocket returned to its original conformation (Figure 5A).

### 2.4. Interaction between RadD and DNA

**Figure 6:**
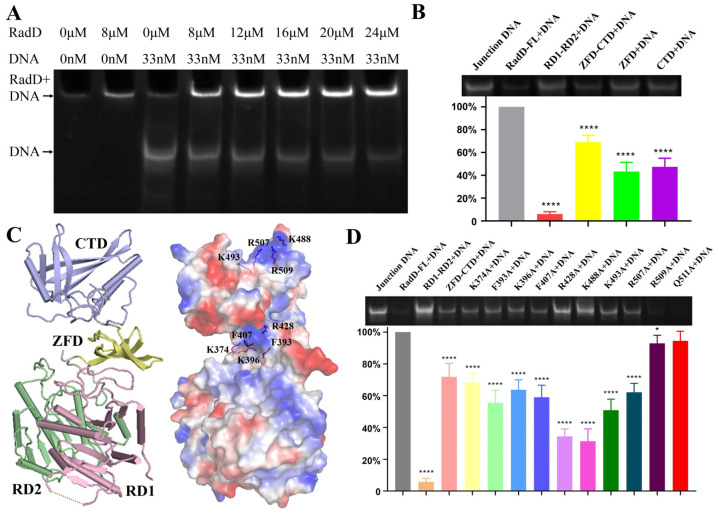
Specific interactions between RadD and branched DNA. (**A**) EMSA assays show that the branched DNA concentration decreases with the increase in RadD concentration. (**B**) EMSA assays and relative value statistics of RadD deletions combined with branched DNA. (**C**) The key amino acids binding to DNA are predicted in accordance with the surface potential diagram of the RadD/ATP structure. (**D**) Mutagenesis analysis of RadD and branched DNA interface residues, as shown by EMSA assays and relative value statistics. Error bars indicate the S.D. of at least three independent experiments, * means *p*-value < 0.1, **** means *p*-value < 0.0001.

RadD interaction with ssDNA dt 100 was reported by Stefanie. H.C. in 2016 [11]. Here, our EMSA assay results indicated that the RadD displayed a high affinity for branched DNA similar to its homologous protein RecQ [24,25] (Figure 6A). Following the structure of RadD/ATP, we designed several truncations to explore the interaction region between RadD and DNA. The results showed that the binding amount by RD1-RD2 was lower (6.0%) compared with that of wild-type (100%), indicating that RD1 and RD2 cannot bind branched DNA. The binding amount by ZFD-CTD, ZFD, and CTD to DNA were 69%, 43%, and 47%, respectively (Figure 6B). The above data verified that the ZFD and CTD of RadD were important binding sites for branched DNA. RD1 and RD2 did not bind to DNA, but their ability to help ZFD and CTD in forming the rigid structure of RadD improved the binding to DNA.

“Grooves” were formed by positively charged amino acid residues on the surfaces of ZFD and CTD domains after observing the surface potential diagram of RadD (Figure 6C). The amino acid residues in these two “grooves” might be the key sites of RadD and junction DNA interaction. Thus, the single-site mutants were designed, and EMSA experiments were performed with single-site mutants and DNA. The results showed that the relative amounts of DNA binding by RadD_R428A_ and RadD_K488A_ were 34% and 31% compared with the RadD, respectively, which indicated that Arg428 on the ZFD and Lys488 on the CTD domains of RadD were the key amino acid residues of RadD and DNA interaction (Figure 6D). In addition, RadD_F393A_, RadD_K396A_, RadD_F407A_, RadD_K493A_, and RadD_R507A_ bound DNA amounts to between 50% and 70% wild-type.

## 3. Discussion

The ATPases work with the energy generated by the hydrolysis of ATP, and many of them share similar domains. This finding is first discovered in RecA, a DNA recombinant repair protein of *E. coli*. Thus, a similar domain is named RD [26]. These ATPases containing RD catalyze the hydrolysis of ATP by a similar mechanism. Combined with the structures of the RadD/ADP and RadD/ATPγS complex and the results of the RadD ATPase assay, we explained the dynamic details of the ATP hydrolysis mechanism of RadD in *E. coli.*

In addition to the conserved Lys37 in Walker A and Arg343 in motif VI making direct contact with the ATP’s β and γ- phosphate, the Gly34 and Gly36 also interact with the ATP, and mutations in these four residues result in RadD protein loss of ATPase activity. The Walker A motif is slightly expanded to accommodate the triphosphate tail, apparently as a consequence of the motion of Ser38 because it interacts with the bound Mg^2+^. In addition to the triphosphate tail, the ATP molecule is also bound to RadD at the adenine. The N7 and N6 of adenine form hydrogen bonds to the side chains of Arg6 and Gln9 in motif Q.

We find that Lys68 plays a unique role in RadD as a switch for ATP binding and hydrolysis. In contrast to apo-RadD, the side chain of Lys68 in motif Ia is shifted away from the active site by 4.7 Å to make room for the ATP molecule and to promote the binding rate of RadD when ATP is bound. The ATPase activity of mutant K68A is three times that of the wild-type, suggesting that Lys68 modulates RadD’s catalytic rate. The conserved Asp117 in the Walker B motif of RadD does not bind Mg^2+^ and acts to polarize the attacking water molecule, which is different from other members of SF [27,28] (Figure S2). Lys37 is located between the γ-phosphate of ATP and Asp117, which are on the same line and form an equilibrium state by the electrostatic attraction (Figure 3B). The side chain of Lys37 shifts away due to the exit of the ADP molecule, and Asp117 will correspondingly move back to its prepolarized attacking water configuration.

Many proteins involved in DNA replication, repair, and recombination can interact with SSB, particularly with the C-terminal of SSB. SSB improves the DNA helicase activity of RecQ or PriA [17,18] and the ATP hydrolysis and DNA-binding activities of RecG [19]. RadD can also interact with SSBct, which increases the ATPase activity of RadD, and a possible working model of RadD in DNA damage repair has been speculated. When encountering RNA polymerase stagnated due to DNA damage, SSB can recruit and activate RadD to help RadD remove RNA polymerase from damaged DNA [10,11,29]. Here we show that the CTD of RadD interacts with SSB, but how SSB and RadD cooperate to function in DNA repair needs further investigation.

RadD, as an SF2 helicase [30], has a high similarity with RecQ, BLM, and RecG proteins in the SF2. Extensive effort has been exerted to detect and characterize the helicase activity by us, however, the RadD helicase activity has not been observed on oligo-based helicase substrates of any structure under any conditions despite the capacity of RadD to bind synthetic replication forks [29].

**Figure 7:**
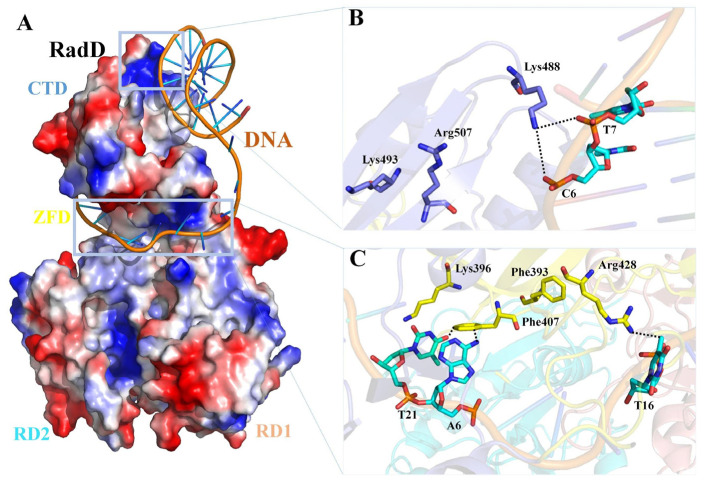
Predictive model of the RadD/DNA complex. (**A**) Surface potential diagram of RadD binding branched DNA. The blue area is shown as the positive charge and the red area is the negative charge, and the branched DNA is shown as a cartoon. (**B**) Interface residues mediating interactions between the RadD-CTD and branched DNA. (**C**) Interface residues mediating interactions between the RadD-ZFD and branched DNA. CTD is shown in blue, ZFD in yellow, DNA in orange, and the bases in cyan.

RadD and RecQ helicase cores have similar domain structures. The main differences are the existence of a unique CTD in RadD and a C-terminal wing helix domain in RecQ. The structural comparison of RadD with RecQ/DNA [31], RecG/DNA [27], and BLM/DNA [32] complexes indicate that RadD has similar catalytic centers with the above three SF2 family proteins, and they both have RD1 and RD2 regions for ATP binding and hydrolysis. The binding regions of DNA in the SF2 helicase family are relatively different. The structural comparisons show that the DNA-binding region of RadD is located at the C-terminal of the protein, which differs from the DNA-binding regions of RecQ, BLM, and RecG (Figure S3). On the basis of the results of biochemical assays of the interaction of RadD with branched DNA, we propose the model of RadD binding DNA using the COOT software [33] combined with the HDOCK server [34]. The branched DNA binds to the CTD of RadD, extending to the horizontal deep groove that forms between the CTD and ZFD (Figure 7A). Phe407, Arg428, and Lys488 interact directly with the branched DNA and are important residues of RadD bound DNA, in agreement with the assays of our biochemical experiments. Although Phe393, Lys396, Lys493, and Arg507 do not interact with DNA, they stabilize RadD and DNA interactions (Figure 7B,C).

In this study, our structural and functional analyses clearly show that RadD consists of two functional regions. The functions of these two regions are independent of each other. RD1 and RD2 are responsible for ATP binding and hydrolysis, and Lys68 and Asp117 play unique roles in the activity of RadD ATPase, unlike other DNA repair proteins in the SF2 family. ZFD and CTD are responsible for nucleic acid binding. Arg428 on ZFD and Lys488 on CTD are key residues for binding to the target DNA. CTD is also the only region of RadD where SSB is bound. Our study provides insight into the mechanism of RadD binding to DNA and ATP hydrolysis by helicase SF2 and helps facilitate the study of RadD function in the DNA damage repair process.

## 4. Materials and Method

### 4.1. Protein Purification

Recombinant RadD and SSB were overexpressed in *Escherichia coli* and purified as previously described [14]. RadD site-specific mutants and truncations were generated from the RadD-pET-28a and the RadD-pGEX-6p-1 plasmid. SSBΔC were generated from the SSB-pET-28a plasmid. All sequences were confirmed by sequencing. All proteins were stored at −80 °C.

### 4.2. Crystallization and Data Collection

Crystals of RadD/ATPγS were obtained by incubating 5 mg/mL RadD and 5 mM ATPγS for 30 min at 20 °C, and then growing with drops containing 2.0 μL of RadD/ATPγS and 2.0 μL of reservoir solution (500 mM NaCl, 18% (*w*/*v*) PEG3350). Crystals of RadD/ATP were obtained by incubating 5 mg/mL RadD and 5 mM ATP for 30 min at 20 °C, and grown under the same conditions as RadD/ATPγS, except that Mg^2+^ ion was added to the solution (500 mM NaCl, 5 mM MgCl_2_, 18% (*w*/*v*) PEG3350). The crystals were flash-frozen by immersion in a reservoir solution supplemented with 10% glycerol followed by flash-cooled into liquid nitrogen. During 100K, X-ray diffraction data collection using the beamline BL19U1 (λ = 0.979 Å) at the Shanghai Synchrotron Radiation Facility (SSRF; Shanghai, China). The diffraction images were indexed and integrated using HKL3000 [35]. The data collection statistics are presented in Table S1.

### 4.3. Structure Determination and Refinement

The structures of RadD/ATPγS and RadD/ADP were solved by the molecular replacement method using PHASER in the PHENIX with one monomer of RadD (PDB code: 6JDE) as the search model at 3.0 Å resolution [36]. Iterative cycles of refinement and manual model building were carried out with PHENIX refinement programs [37] and COOT [33] at 20-3.0 Å and 20-2.7 Å resolution, respectively. All structural images were drawn using PyMOL (http://www.pymol.org/). Detailed crystallographic statistics are shown in Table S1.

### 4.4. ATPase Assay

ATPase activity assay was performed using the ATPase Assay Kit (BioAssay Systems, Hayward, CA, USA). Purified RadD (0.4 μM) was incubated in 40 μL ATPase assay buffer (20 mM Hepes 7.5, 150 mM NaCl, 3 mM MgCl_2_) supplemented with 0.25, 0.33, 0.5, 1.0, 2.0, 3.0, 5.0, 7.0, 10.0 mM ATP at 20 °C for 30 min, respectively. The reaction was terminated with 200 μL malachite green solution for another 30 min. The absorbance was measured at 620 nm in a 96-well plate reader Ensprie (PerkinElmer, Waltham, MA, USA). Calibration of free phosphate (Pi) concentration was carried out with a Standard Solution (1 mM phosphate). Measurements were performed in triplicate. Data analysis was performed in Origin.

### 4.5. GST-Pull Down Assays

An amount of 5 μM RadD native or truncations with GST tag was incubated with 50 μM SSB in 200 μL binding buffer (20 mM Tris-HCl 8.0, 300 mM KCl, 2 mM DTT) for 30 min at 20 °C, respectively. After centrifuging (12,000 rpm, 10 min), the supernatant was incubated with 20 μL (wet volume) GST beads (GE, Boston, MA, USA), which was equilibrated by binding buffer in advance, for another 30min at 20 °C. GST beads were washed three times with binding buffer and the beads were eluted with 50 μL binding buffer supplement with 20 mM reduced GSH. Proteins bound to the beads were analyzed by 15% SDS-PAGE and visualized by Coomassie staining.

### 4.6. Electrophoretic Mobility Shift Assays (EMSA)

Branched DNA substrates: oligos were ordered from Invitrogen Inc. (USA) with the following sequences by HPLC.

Holly junction DNA:

oligo1: CCGCTACCAGTGATCACCAATGGATTGCTAGGACATCTTTGCCCACCTGCAGGTTCACCC

oligo2: TGGGTGAACCTGCAGGTGGGCAAAGATGTCCTAGCAATCCATTGTCTATGACGTCAAGCTC

oligo3: GAGCTTGACGTCATAGACAATGGATTGCTAGGACATCTTTGCCGTCTTGTCAATATCGGC

oligo4: TGCCGATATTGACAAGACGGCAAAGATGTCCTAGCAATCCATTGGTGATCACTGGTAGCGG

Three-way junction replication forks were generated from oligo1, oligo2, and oligo5 with the sequence: GAGCTTGACGTCATAGACAATGGATTGCTATAGCAATCCATTGGTGATCACTGGTAGCGG

Annealing reactions to create branched DNA substrates were carried out by adding equimolar oligo amounts in buffer (10 mM Tris HCl 7.5, 100 mM NaCl, 10 mM MgCl_2_), with an initial temperature of 95 °C, then reducing the temperature by 10 °C every 5 min, until the temperature drops to 25 °C. Keep for 10 min. The refolded DNA was purified using Superdex 200 10/30 column by AKTA Purifier (GE, MA, USA), the buffer is 10 mM Tris HCl 7.5, 100 mM NaCl (Figure S4).

EMSA: a certain concentration gradient (0–20 μM) of RadD protein was associated with 33 nM HJ mixed incubation of 37 °C, 30 min. The binding buffer is 20 mm Bis-Tris 6.5, 50 mM NaCl, 15% glycerol, and 10 mM β-Mercaptoethanol. The incubated samples were directly run with an 8% non-denatured PAGE gel, and the electrophoresis buffer was 0.5 × TBE, electrophoresis condition 100 V, 80 min. Gels were imaged using the ChemiDocTMXRS+ Imaging System (BIO-RAD). Quantitative calculation of the gray value in the gel image is calculated by using Quantity One software and statistical mapping by GraphPad Prism software. All data are representative of at least three independent experiments. A *p*-value < 0.05 was considered statistically significant.

## Data Availability

The structure factors and coordinates have been deposited in the Protein Data Bank; RadD/ATPγS PDB: 8H5Z; RadD/ADP PDB: 8H5Y.

## Supplementary Materials

The following supporting information can be downloaded at: https://www.mdpi.com/article/10.3390/ijms24010741/s1.
