# Supplementary material for: Biochemical and Structural Analyses Shed Light on the Mechanisms of RadD DNA Binding and Its ATPase from Escherichia coli
Source: Int J Mol Sci. 2023 Jan 1;24(1):741. doi: 10.3390/ijms24010741 (PMC9821108; doi:10.3390/ijms24010741)
Supplement: Supplementary file 1 [file ijms-24-00741-s001.zip › ijms-2064957-supplementary.pdf]

Supporting Information

Biochemical and Structural Analyses Shed Light on the Mechanisms of RadD DNA Binding and Its ATPase from *Escherichia coli*

Li-Fei Tian[1], Xiaolin Kuang[1], Ke Ding[1, 2], Hongwei Gao[1, 2], Qun Tang[1], Xiao-Xue Yan[1], Wenqing Xu[1,3]

[1]National Laboratory of Biomacromolecules, CAS Center for Excellence in Biomacromolecules, Institute of Biophysics, Chinese Academy of Sciences, Beijing 100101, P. R. China.

[2]College of Life Sciences, University of Chinese Academy of Sciences, Beijing 100049, China.

[3]School of Life Science and Technology, ShanghaiTech University, Shanghai, China.

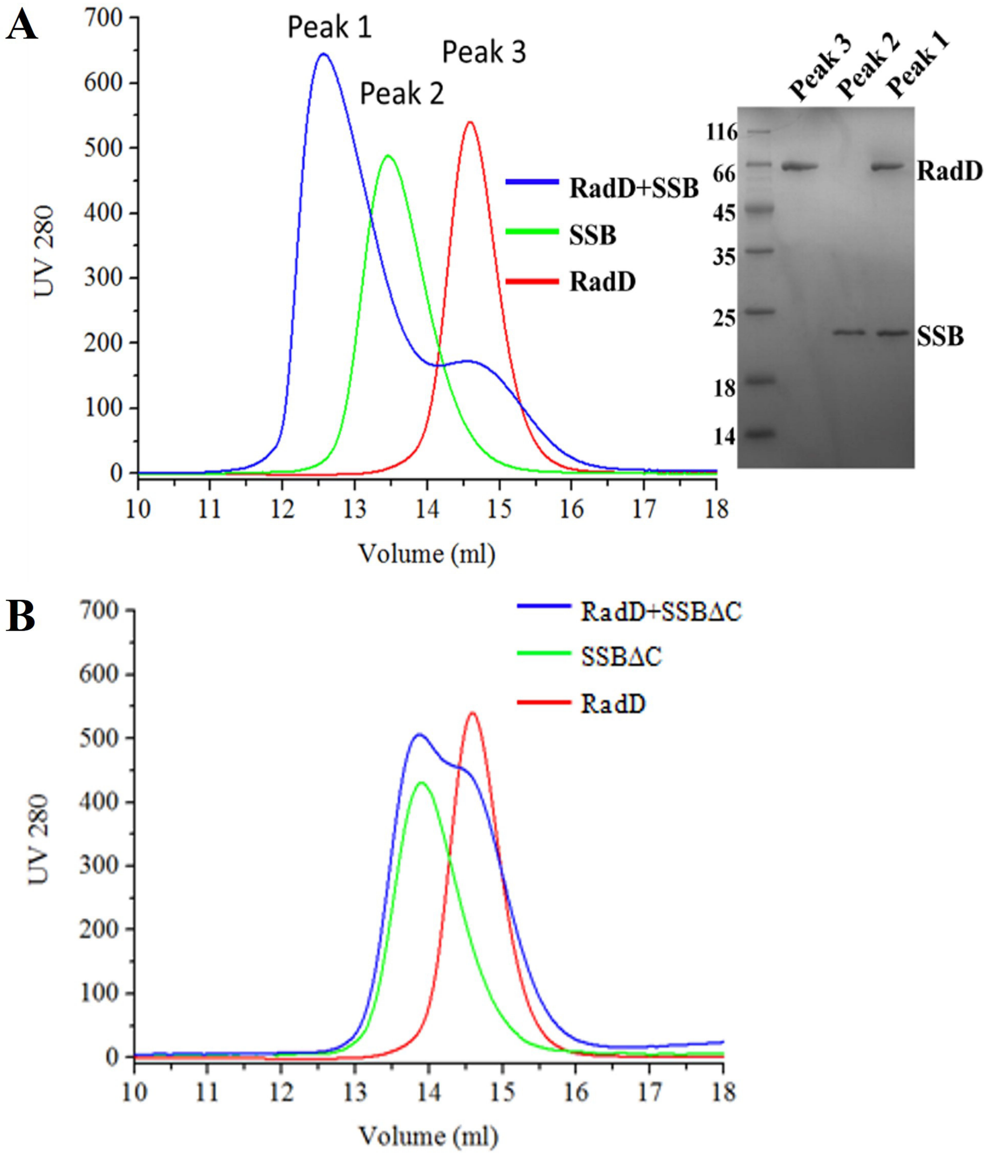

Figure S1. Interactions between RadD and SSB *in vitro* by size-exclusion chromatography assay. (A) Co-migration of full-length RadD and full-length SSB on size-exclusion chromatography. The peaks of RadD and SSB alone are 13.5-15.5 mL and 12.5-14.5 mL, respectively. After mixing and incubating, the peak position of RadD/SSB is advanced to 12.0-14.0 mL, indicating the formation of a stable complex. (B) The peak of SSBΔC was 13.0-15.0 ml, After mixing and incubating, the peak position on the molecular sieve did not Co-migration. Which indicates that RadD and SSBΔC has no stable interaction. So, the SSBCt is indispensable in RadD and SSB interactions.

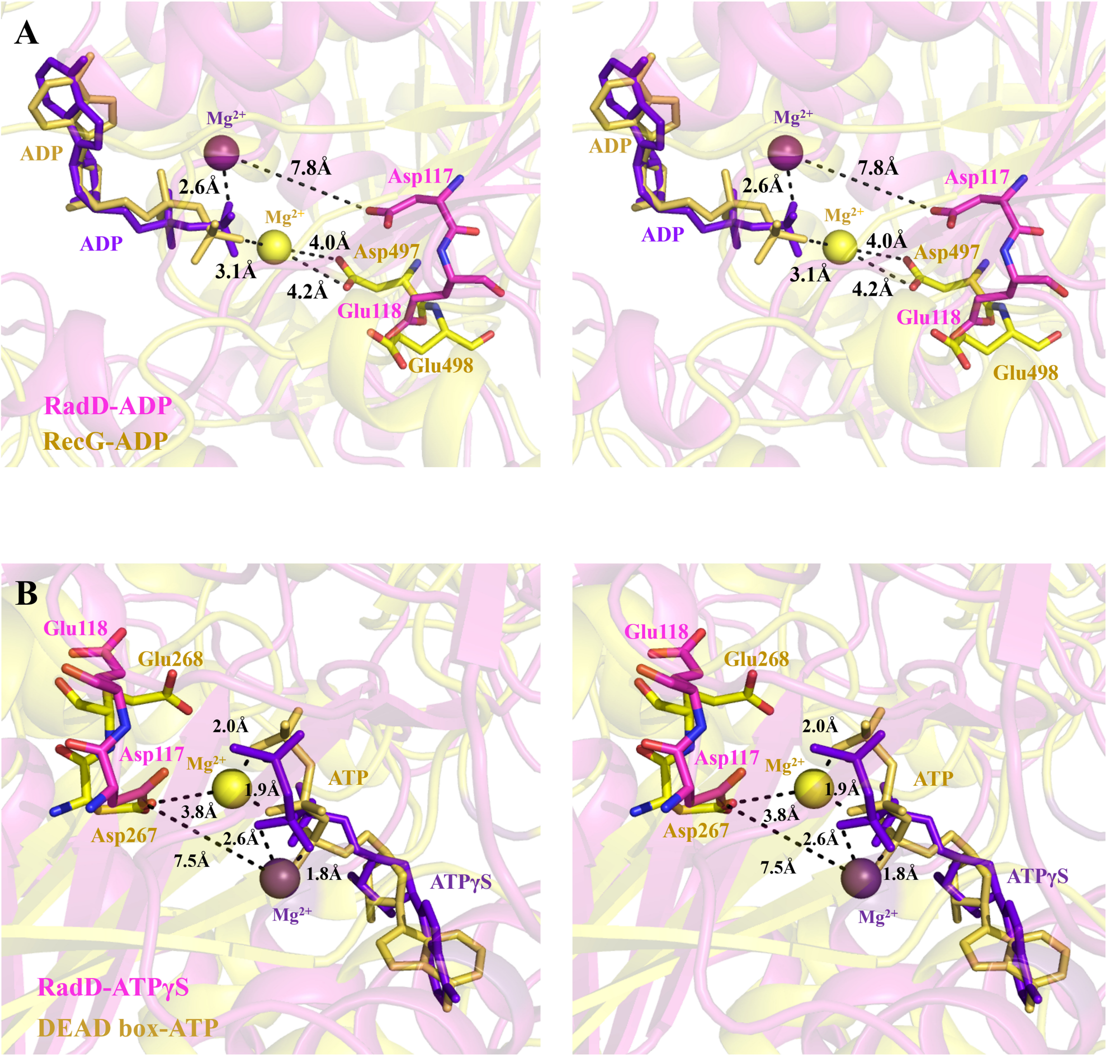

Figure S2. Conservative Asp117 on the Walker B motif of RadD is not in direct contact with the bound Mg^{2+} ion, unlike other members of SF. (A) Comparison of RadD and RecG (PDB: 1GM5) in active center, Asp497 corresponding to Asp117 of RadD is closer to Mg^{2+}, and interacts directly with Mg^{2+}. (B) Comparison of RadD and DEAD box protein (PDB: 3I5X) in active center, Asp267 corresponding to Asp117 of RadD is closer to Mg^{2+}, and interacts directly with Mg^{2+}. RadD/ATP or ADP is showed in purple, RecG/ADP and DEAD box protein/ATP in yellow, Mg^{2+} of RedD and RecG (DEAD box protein) in violet and brown, respectively.

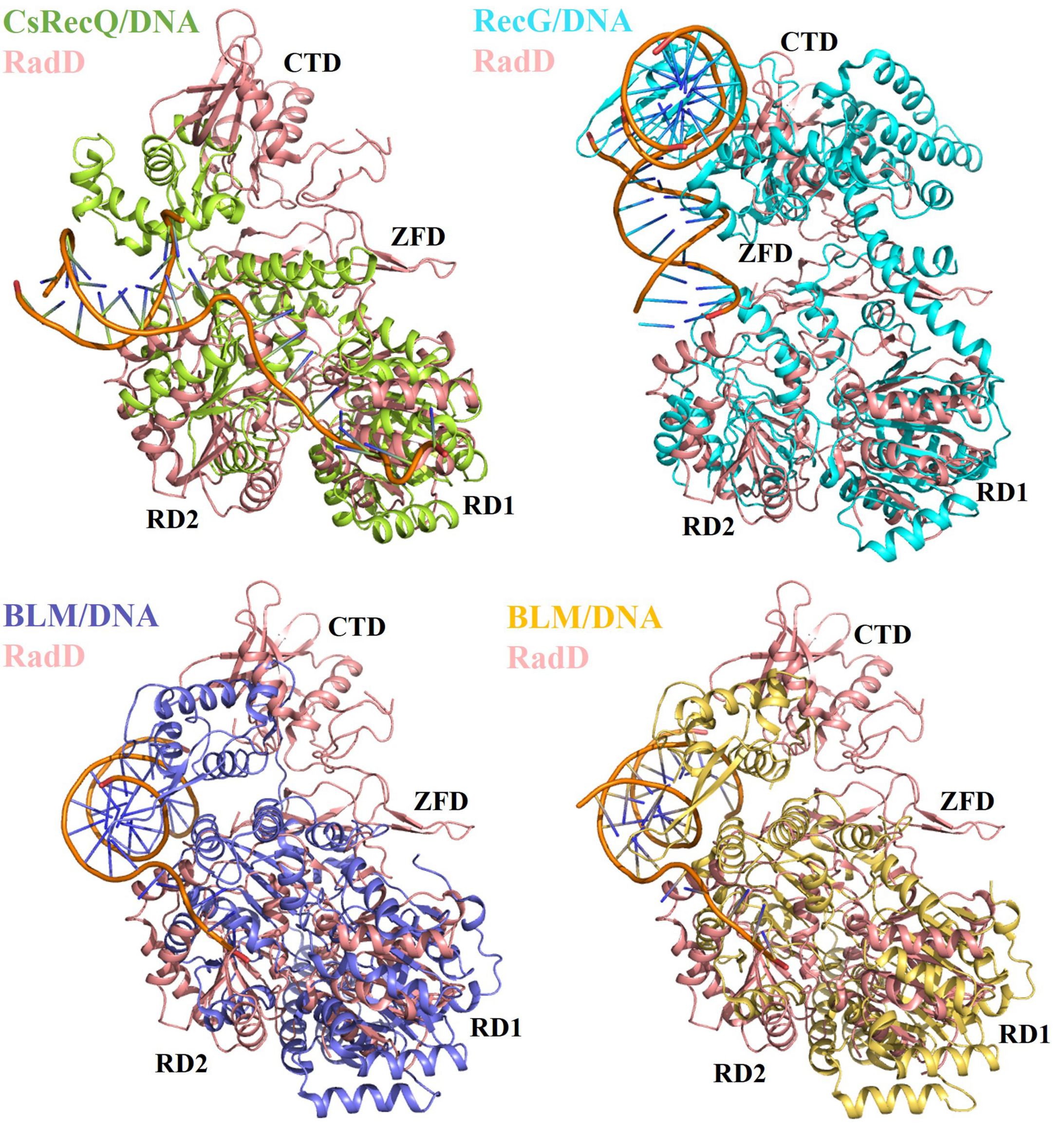

Figure S3. Structural comparisons between RadD and RecQ/DNA (PDB: 4TMU4), RecG/DNA (PDB: IGM5), BLM/DNA (PDB: 4CGZ4) and BLM/DNA (PDB: 4O3M) complexes in SF2 helicase family. RadD is represented in wheat, RecQ in olive, BLM in blue and yellow. The helicase center (RD1 and RD2) of RadD is similar to RecQ, RecG and BLM. The main difference is a C-terminal wing helix domain in RecQ and a unique CTD in RadD. Structural comparison shows that the DNA binding site is located at the CTD of RadD, which is quite different from RecQ, RecG and BLM.

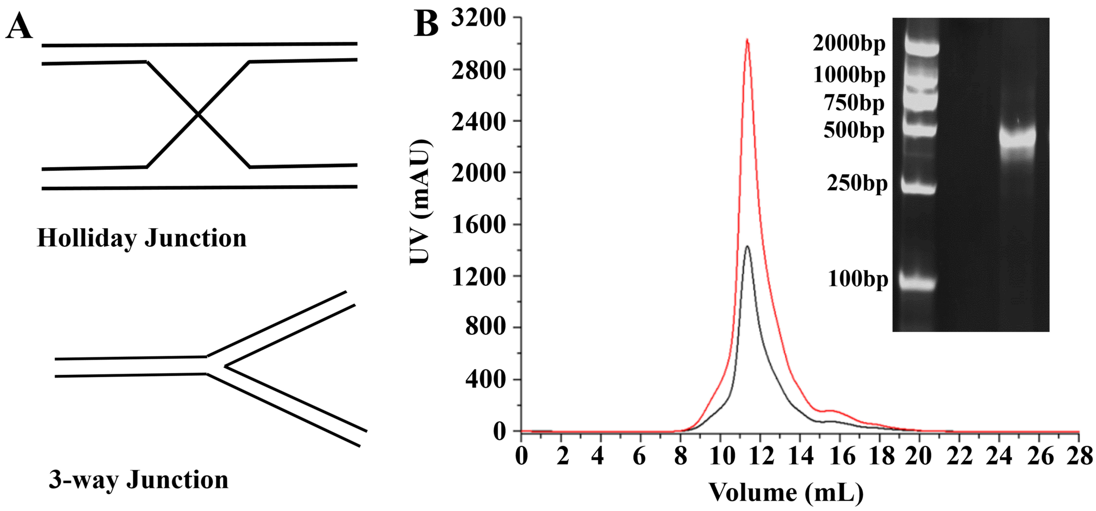

Figure S4. Preparation of branched DNA. (A) The schematic diagram of 3-way junction replication forks and Holly junction DNA. (B) The branched DNA was purified using Superdex 200 10/30 column.

Table S1. X-ray Data collection and refinement statistics

**Data collection**	**RadD-ADP**	**RadD-ATPγS**
Space group	$P2_1$	$P2_1$
Cell dimensions		
a, b, c (Å)	82.09, 78.77, 110.23	82.59, 79.17, 109.82
α, β, γ (°)	90, 99.56, 90	90, 98.84, 90
Wavelength (Å)	0.979	0.979
Resolution (Å)	50.0-2.70 (2.77-2.70)	50.00-3.00 (3.05-3.00)
R_{pim} (%)	4.8(20.4)	8.1(39.8)
I / σI	14.5(4.2)	18.4(2.3)
CC1/2	99.3(94.5)	73.7(76.7)
Completeness (%)	99.9(100.0)	99.8(99.9)
Redundancy	6.8(6.4)	6.6(6.4)
**Refinement**		
Resolution (Å)	20-2.70	20-3.00
No. reflections	37982	28021
R_{work}/R_{free}[a,b] (%)	22.3/26.9	23.94/28.13
No. atoms	1354	1106
R.m.s deviations		
Bond lengths (Å)	0.012	0.012
Bond angles (°)	1.465	1.740
Ramachandran plot (%)		
Most Favorable	91.4	91.3
allowed	8.6	8.7
Outliers	0	0

Values in parentheses are for highest-resolution shell.

[a] $R_{work} = \Sigma_{hkl} |Fo(hkl) - Fc(hkl)|/\Sigma_{hkl} Fo(hkl)$.

[b] R_{free} was calculated for a test set of reflections (5%) omitted from the refinement.
